# Survival Analysis of Patients with Colorectal Cancer Undergoing Combined Treatment: A Retrospective Cohort Study

**DOI:** 10.34172/jrhs.2023.107

**Published:** 2023-03-30

**Authors:** Daem Roshani, Ghobad Moradi, Mohammad Aziz Rasouli

**Affiliations:** ^1^Social Determinants of Health Research Center, Research Institute for Health Development, Kurdistan University of Medical Sciences, Sanandaj, Iran; ^2^Department of Epidemiology and Biostatistics, Faculty of Medicine, Kurdistan University of Medical Sciences, Sanandaj, Iran; ^3^Clinical Research Development Unit, Kowsar Hospital, Kurdistan University of Medical Sciences, Sanandaj, Iran

**Keywords:** Survival, Colorectal cancer, Cancer treatment, Retrospective, Study design, Cohort

## Abstract

**Background:** If colorectal cancer (CRC) is diagnosed in the early stages, the patients will have higher survival rates. Although some other factors might affect the survival rate, the type of treatment available based on existing health and therapeutic facilities is extremely important as well. Accordingly, this study aimed to explore the best type of treatment for CRC patients.

**Study Design:** This study employed a retrospective population-based cohort design.

**Methods:** The data of 335 patients with CRC in Kurdistan province were collected through a population-based cancer registry system from March 1, 2009 to 2014. Demographic and clinical-pathologic data of the patients were gathered through their medical records, pathology reports, and reference to patients’ homes. The survival rate was calculated using the Kaplan-Meier curve, log-rank test, and univariate and multivariate Cox regression. The data were analyzed using Stata 14 software.

**Results: ** In this study, the mean age±standard deviation at diagnosis was 61.7± 1.05 in men and 60.5± 1.12 in women, respectively, and 203 (60.5%) patients were males. There was less mortality rate among the patients who received both surgical and chemotherapy treatments compared to those who did not receive any treatment (Hazard ratio [HR]=0.57, 95% CI: 0.24-0.93).

**Conclusion:** When CRC patients are treated using both surgical and chemotherapy treatments, they will exhibit a higher survival rate. Therefore, it is suggested to use both treatments for CRC patients.

## Background

 Approximately 1.2 million people suffer from colorectal cancer (CRC), the third most deadly cancer worldwide.^1^ CRC has been ranked as the fourth cancer among Iranian males.^2^ Cancer survival is an indicator of the overall effectiveness of health services in the management of patients.^3^ The 5-year survival rate of individuals with CRC survival rate is different across countries in the world. Meanwhile, the 5-year survival rate for CRC is 64%, but it decreases to 12% for metastatic CRC; thus, further research is needed to develop effective approaches for medical intervention.^4^ In a review study, the 1, 3, and 5-year survival rates of CRC in Iran were calculated as 85%, 65%, and 52%, respectively.^5^ These different rates of survival demonstrate the heterogeneity of studies in terms of design, population, tumor stage, socioeconomic status, pathologic factors, and the treatment types.^3,6^

 CRC patients’ prognosis depends directly on the diagnosis time and its stage.^7,8^ A combination of various types of treatments is used in the advanced stages of the disease. Standard treatment methods used for CRC patients are different based on the tumor size, stage of the tumor, and the patient’s physical characteristics.^9^

 Due to advances in primary and adjuvant therapies, survival time in CRC has improved. Typically, an ideal treatment for CRC is to achieve the complete removal of the tumor and metastases, which most often requires surgical intervention.^10,11^ Appropriate surgery is recognized as the most important aspect of CRC treatment and as a necessary curative treatment modality for CRC.^12-14^ However, the inclusion of other modalities in the treatment of CRC (e.g., chemotherapy and radiotherapy) can reduce the probability of disease recurrence.^15,16^ Surgery and chemotherapy have long been the first choices for cancer patients. However, the prognosis of CRC has never been satisfying, especially for patients with metastatic lesions. Although treatment advances have significantly improved CRC survival, clinical outcomes vary widely among patients with tumors diagnosed at the same tumor, node, and metastasis stage.^17,18^

 The effects of different types of treatments on CRC have been reported variably in different studies. The results of different studies on the stage, type of treatment, and the patient’s survival rate showed improvement in treatment worldwide in recent decades.^14,19^ CRC treatment procedure including screening, surgery, chemotherapy, and radiotherapy has improved in the last decades, and the 5-year survival for the patients has, at least, increased from 51% to 65% over the last 30 years.^20,21^ Increasing evidence demonstrated an increase in CRC patients survival time at an international level, suggesting that some changes in quality and the type of the treatments might have led to the increase in the survival rate.^22,23^

 This study strived to investigate the effects of different types of treatments, with regard to other demographic and clinical factors, on estimating the 5-year survival rate of CRC patients in Kurdistan province.

## Methods

###  Data source

 This is a retrospective cohort study in which the data of 335 CRC patients in Kurdistan province (west of Iran) were investigated in a population-based study. Patients’ data were collected from March 2009 to 2014 using the cancer registry system. Diagnosis and registration of the patient’s data were encoded and extracted based on international classification diseases (ICD10), anatomic location of the colon (C18), rectosigmoid (C19), and rectum (C20) cancers.

 To analyze data, we applied survival models for right-censored time-to-event data that are more adequate than those in classical regression models in two ways. First, survival models are among the most appropriate statistical methods considering censored time-to-event data. Secondly, they include the duration of an event.^24,25^ The CRC survival was calculated from the date of diagnosis to the date of cancer death or the end of follow-up (Cutoff date: October 2015). Then, demographic data of the patients including their age at the time of diagnosis, gender, address, insurance status, history of smoking, socio-economic status (based on welfare status using principal component analysis), and cancer family history were gathered through patients’ medical records and referring to their houses as well. Moreover, pathologic and clinical data of the patients were gathered using their pathology reports, medical records, and cancer registry system. The patients’ treatment status was determined using their medical records and interviewing with the patients themselves or one of their family members on the treatment procedure. Further, their standard treatment procedure including surgery, chemotherapy, and radiotherapy alone or in combination was determined using the patients’ medical records. They were assured that their demographic, pathological, and clinical information as well as their medical records would remain confidential.

###  Statistical analysis

 In this study, the follow-up time was defined as the date of diagnosis until the date of survival, death, and censoring (Cutoff date: October 2015). The Kaplan-Meier method was used to determine the observed cumulative survival probability over time by calculating the proportion of surviving after being diagnosed with CRC. Then, five-year survival probabilities for CRC were developed to compare the overall survival probability across the different treatment site categories.

 The correctness of the model fitted to the observations was determined using the residual Martingale plot.^24,26,27^ Hazard ratios (HRs) for CRC survival were calculated using the Cox proportional hazard model to adjust for age at diagnosis, socioeconomic status, and types of treatment.

 The variables with significant levels less than 0.1 (*P* < 0.1) were entered into the model, and the HR and 95% confidence interval (CI) were determined for estimating the effect of variables on the death risk resulting from CRC. Then, the stepwise multiple regression method was used to analyze variables that independently had a significant relationship with the survival rate. Data analysis was conducted using Stata 14 (Stata Corp, College Station, TX).

###  Ethics statement

 The present study was performed under the tenets of the Declaration of Helsinki. Ethical feasibility was obtained from the Ethics Committee of Kurdistan University of Medical Sciences (IR.MUK.REC.1394.36). This study is a part of a student’s master’s thesis in epidemiology.

## Results

 In this study, the mean age ± standard deviation at diagnosis was 61.7 ± 1.05 in men and 60.5 ± 1.12 in women, and the incidence of this disease was higher among men (60.5%). Regarding the type of treatment, surgery, chemotherapy, and radiotherapy were used in 43 (60%), 59 (17.5%), and 23 (6.8%) patients. The 1, 3, and 5-year survival rates of CRC in patients who used a combination of treatment (surgery and chemotherapy) were calculated as 89.2%, 73.1%, and 55.8%, respectively. Other prognostic factors are presented in Table 1.

**Table 1:** Characteristics of the study population and 1, 3, and 5-years CRC survival [%] for Kurdistan province residents diagnosed during 2009-2014 (N = 335)

**CRC-survival (%)**	**Number**	**Percent**	**1 Year**	**3 Year**	**5 Year**	***P*** ** value **
Gender						0.296
Male	203	60.5	85.9	52.1	33.1	
Female	132	39.5	89.3	73.3	32.0	
Age at diagnosis (year)						0.001
≤ 50	82	24.4	90.1	79.4	65.7	
51-64	116	34.6	85.2	63.7	45.4	
≥ 65	137	41.0	78.4	49.9	14.6	
Residential area						0.023
Rural	108	32.3	85.0	48.2	22.2	
Urban	227	67.7	88.3	60.3	39.3	
Insurance						0.125
No	28	8.4	61.7	38.2	34.1	
Yes	307	91.6	90.6	59.5	30.4	
Smoking						0.017
No	253	75.5	88.0	58.9	36.1	
Yes	82	24.5	85.0	47.6	23.8	
Socioeconomic status						0.002
Poor	105	33.5	90.7	70.8	44.7	
Moderate	104	33.2	89.4	51.9	30.6	
Rich	104	33.2	72.7	54.3	8.4	
Family history of CRC						0.556
No	293	87.5	87.5	67.5	37.3	
Yes	42	12.5	85.4	54.3	32.0	
Comorbidity						0.026
No	205	61.2	87.2	59.1	41.6	
Yes	130	38.8	87.4	52.0	23.3	
Tumor location						0.717
Colon	201	60.0	87.5	54.6	34.3	
Rectum	94	28.1	84.6	58.7	35.4	
Recto sigmoid	40	11.9	92.5	61.0	27.4	
Tumor node metastasis stage						0.085
II	68	20.3	89.7	74.3	40.4	
III	168	50.1	86.7	52.1	30.8	
Histologic type						0.599
Adenocarcinoma	308	91.9	86.8	56.5	32.4	
Mucinous/Other	27	8.1	92.5	51.4	41.3	
Tumor grade						0.001
Well differentiated	99	29.5	95.8	69.8	54.4	
Moderately differentiated	106	31.6	85.7	61.8	42.1	
Poorly differentiated	86	25.7	81.2	51.2	23.5	
Undifferentiated/anaplastic	44	13.2	83.7	28.4	4.1	
Treatment						0.001
None	10	3.0	53.2	33.2	00.0	
Surgery	42	12.5	85.8	44.2	22.1	
Chemotherapy	59	17.5	86.0	46.5	23.6	
Radiotherapy	23	6.8	85.2	62.1	24.1	
Surgery + radiotherapy	86	25.6	88.1	73.2	46.5	
Surgery + chemotherapy	94	28.0	89.2	73.1	55.8	
Surgery + chemotherapy + radiotherapy	21	6.6	81.3	41.2	30.4	

*Note.* CRC: Colorectal cancer.

**Figure 1:**
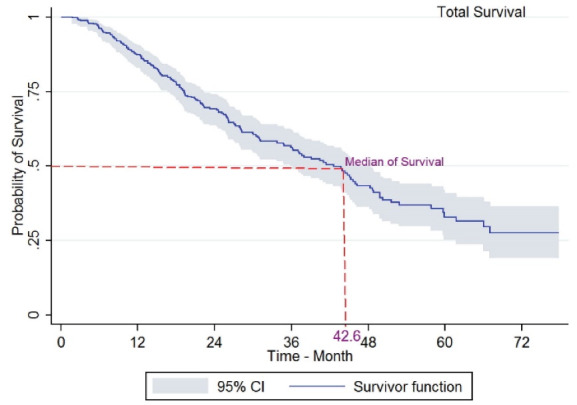
Five-year survival of patients diagnosed with colorectal cancer in Kurdistan province (2009–2014) (Kaplan–Meier)

 The 1, 3, and 5-year survival rates were 87%, 57%, and 33%, respectively. Further, the median survival time was 42.6 months (Figure 1), and the median survival was higher among women. Forty-four patients (13.3%) were diagnosed with undifferentiated anaplastic tumor grade, for which the Log-rank test showed a significant difference (*P* < 0.001).

**Figure 2:**
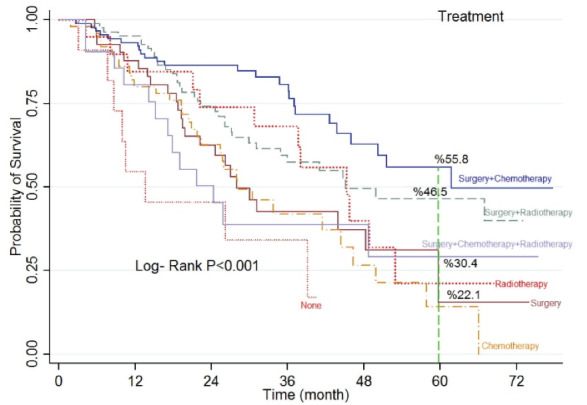
Kaplan–Meier curves of colorectal cancer-specific survival regarding treatment

 There was a significant statistical difference among the type of treatments received by the patients (*P* < 0.001). The results indicated that 3.6% of the patients do not receive any kind of treatment, and the survival rate is the least in this group (13.6 months). Moreover, the five-year survival rate in patients who received both surgery and chemotherapy was 55.8%, and it was 46.5% for the patients who received surgery and radiotherapy (Table 1 and Figure 2).

 Univariate and multiple Cox regression analysis for the patients, based on the type of treatments they received, revealed that HR and mortality rates for the patients who received both surgery and chemotherapy are lower those for the patients who did not receive any treatment (HR = 0.57, 95% CI: 0.24-0.93). However, univariate regression analysis showed that the patients who received surgery and radiotherapy have a higher survival rate, but multiple regression analysis did not exhibit a significant difference in this group’s survival time (HR = 0.64, 95% CI: 0.22-1.33). Furthermore, there was not a significant difference in the survival rate of patients who received only surgery, chemotherapy, or radiotherapy (*P* > 0.05), as depicted in Table 2.

**Table 2:** Univariate and multiple regression analysis for risk of CRC death (all CRCs)

**Variables**	**Univariate HR (95%CI)**	***P*** ** value**	**Multiple HR (95%CI)**	***P*** ** value**
Gender				
Female	1.00			
Male	1.18 (0.86,1.61)	0.297	Not included	
Age at diagnosis (y)				
≤ 50	1.00		1.00	
51-64	1.75 (1.03,2.98)	0.024	1.50 (0.80,2.82)	0.201
≥ 65	3.60 (2.20,5.90)	0.001	2.78 (1.53,5.06)	0.001
Residential area				
Rural	1.00		1.00	
Urban	0.69 (0.51,0.95)	0.037	0.98 (0.66,1.41)	0.113
Insurance				
No	1.00			
Yes	0.96 (0.69,1.83)	0.134	Not included	
Smoking				
No	1.00		1.00	
Yes	1.49 (1.07,2.09)	0.024	1.40 (0.95,2.07)	0.163
Socioeconomic status				
Poor	1.00		1.00	
Moderate	0.70 (0.48,1.02)	0.134	0.82 (0.53,1.26)	0.808
Rich	0.43 (0.29,0.65)	0.001	0.47 (0.29,0.78)	0.022
Family history of CRC				
No	1.00			
Yes	0.87 (0.54,1.39)	0.242	Not included	
Comorbidity				
No	1.00		1.00	
Yes	1.41 (0.48,1.02)	0.0.06	1.14 (0.79,1.63)	0.549
Tumor location				
Colon	1.00		Not included	
Rectum	1.05 (0.72,1.51)	0.265		
Recto sigmoid	0.84 (0.52,1.35)	0.324		
Tumor node metastasis stage				
II	1.00		1.00	
III	1.44 (0.94,2.27)	0.412	1.22 (0.74,2.01)	0.317
Histologic type				
Adenocarcinoma	1.00		Not included	
Mucinous/other	0.84 (0.44,1.59)	0.298		
Tumor grade				
Well differentiated	1.00		1.00	
Moderately differentiated	1.44 (0.91,2.29)	0.236	1.57 (0.93,2.66)	0.189
Poorly differentiated	2.04 (1.30,3.20)	0.001	2.61 (1.55,4.38)	0.002
Undifferentiated/anaplastic	3.48 (2.14,5.67)	0.001	3.57 (2.10,6.22)	0.001
Treatment				
None	1.00		1.00	
Surgery	0.69 (0.52,1.14)	0.689	0.77 (0.63,1.55)	0.574
Chemotherapy	0.58 (0.49,1.72)	0.523	0.76 (0.28,2.07)	0.612
Radiotherapy	0.64 (0.53,2.01)	0.673	0.75 (0.30,1.85)	0.534
Surgery + radiotherapy	0.47 (0.41,0.96)	0.042	0.64 (0.22,1.33)	0.184
Surgery + chemotherapy	0.38 (0.22,0.69)	0.002	0.57 (0.24,0.93)	0.039
Surgery + chemotherapy + radiotherapy	0.91 (0.97,2.15)	0.432	0.99 (0.35,2.94)	0.397

*Note.* CRC: Colorectal cancer; HI: Hazard ratio; CI: Confidence interval.

## Discussion

 The results of this study showed that the survival rate of patients who received both surgery and chemotherapy was higher than that of the patients with no treatment or only one type of treatment. In addition, univariate regression analysis showed a lower HR in patients who received surgery and radiotherapy, but multiple regression analysis did not show any significant difference in this group. Moreover, the median survival time for the patients who did not receive any type of treatment was lower compared to other patients.

 The effect of types of treatment on patients’ survival is a matter of controversy among different studies. In a systematic review and meta-analysis on the survival rate of colon and rectum cancer in Iran, the results indicated that the 5-year survival rate of CRC after surgery is 64%.^28^ Studies have found that patients with CRC who receive treatment or surgery may have better overall survival. Results of Mehrabani and Almasi-Hashiani’s study in Iran^29^ reported no significant relationship between the type of received treatment by the patients (surgery, chemotherapy, radiotherapy, and a combination of them) and their survival times, but the mortality rate was higher in patients with no treatment. Moreover, a study carried out by Asghari-Jafarabadi et al in Iran^30^ showed that the mortality rate in patients who received radiotherapy, chemotherapy, and immunotherapy was 2.3 times greater than that in the patients whose initial treatment was surgery, which can be due to the tumor cells’ characteristics. Likewise, another study in Iran revealed that those patients who received chemotherapy after surgery have a lower mortality rate (*P* = 0.018).^31^

 Laohavinij et al^32^ in Thailand found that chemotherapy after surgery indicates a good prognosis for CRC patients. Furthermore, Folkesson et al,^33^ in a study in Sweden, showed that the survival rate of patients who received both surgery and radiotherapy was higher than that of patients who only received surgery. In a study in Denmark, the survival rate of patients with colon cancer who underwent surgery increased from 8% to 15% in the colon, and it increased from 13% to 19% in patients with rectal cancer.^34^ The results of another study indicated that primary tumor resection combined with postoperative chemotherapy in patients with stage IV colon cancer with unresectable metastases was associated with increased long-term survival compared with other treatment options.^35^

 Adjuvant chemotherapy was less beneficial in patients with stage II CRC cancer compared to those with stage III tumor,^11^ suggesting that it might achieve better disease-free survival, and overall survival may be achieved as the former group typically had a better prognosis with a 5-year survival rate of approximately 80% based on the results of clinical trial studies.^36,37^ One of the reasons for less survival rate in patients who only received chemotherapy or surgery in Kurdistan province can be that they were not able to pay for the high cost of chemotherapy and surgery. Moreover, low economic status, low income because of low-paid jobs, and no supplementary insurance to cover all costs can be other reasons in this regard.^38,39^

 Better socioeconomic status is known to be influential on the patients’ survival rate. Multiple Cox regression analysis displayed that patients with better economic status have higher survival rates. Patients’ lower socioeconomic status was related to their jobs, incomes, and their less accessibility to medical facilities in rural areas and small towns which have led to the late diagnosis of CRC in its advanced stages, an increase in the treatment costs, and a decrease in their survival rate. However, the increase in the Spanish, Japanese, and Chinese patients’ mortality rate who live in the USA did not exhibit any significant difference with regard to their socioeconomic status.^40^ Studies have indicated that patients with higher socioeconomic status have better health awareness and are more involved in cancer screening, but patients with low socioeconomic status are less likely to receive cancer screening and adjuvant treatments such as radiotherapy or chemotherapy and have a greater risk of death.^41^ At the same time, patients who received both chemotherapy and surgery seem to have a better socioeconomic status; therefore, they were able to go to private hospitals in larger cities with better treatment facilities that meet higher health standards.

 Early diagnosis, progressive and helping treatment in the early stages of cancer, and valid tumor grade classification are effective factors in the patients’ survival rate. Treatment quality and availability, better socioeconomic status, diagnosis in the early stages of the disease, and the type of treatment are influential factors on the CRC patients’ survival rate, and clinical trials or meta-analysis studies can thoroughly investigate the outcomes of the type of the treatment based on different stages of the disease. In addition, as the factors underlying treatment delay were not included in this study, we believe that future studies examining this area may be beneficial.

**Highlights**

- The 1, 3, and 5-year survival rates were 87%, 7%5, and 33%, respectively, and the median survival time was 42.6 months.
- The 1, 3, and 5-year survival rates of colorectal cancer in patients who used a combination of treatment (surgery and chemotherapy) were calculated as 89.2%, 73.1%, and 55.8%, respectively.
- There was not a significant difference in the survival rate of the patients who received only surgery, chemotherapy, or radiotherapy.

## Conclusion

 When CRC patients are treated using both surgical and chemotherapy treatments, they will have higher survival times. Therefore, it is suggested to use both treatments for CRC patients. A combination of surgery and chemotherapy can enhance CRC patients’ survival time, and it is suggested to increase the patients’ accessibility to these treatments.

## Acknowledgments

 We would like to thank the staff of the center for cancer registry at the Health Deputy Department, the Kurdistan University of Medical Sciences, Sanandaj, Iran.

## Competing Interests

 The authors have no conflict of interests to declare for this study.

## Funding

 None.
